# Factors associated with probable cluster of Leptospirosis among kennel workers in Abuja, Nigeria

**DOI:** 10.11604/pamj.2013.16.144.3529

**Published:** 2013-12-19

**Authors:** Emmanuel J Awosanya, Patrick Nguku, Akin Oyemakinde, Olutayo Omobowale

**Affiliations:** 1 Department of Veterinary Public Health and Preventive Medicine, Faculty of Veterinary Medicine, Abadina, University of Ibadan, Agbowo, Ibadan, Oyo State, Nigeria; 2 Nigeria Field Epidemiology and Laboratory Training Programme, 50 Haile Selassie Street, Asokoro, Abuja, Nigeria

**Keywords:** Dog, wounds, sewage, kennel, leptospirosis, risk, infection, zoonosis, workers, Nigeria

## Abstract

**Introduction:**

Between February and October 2009 an increase in morbidity and mortality in dogs in a national kennel in Abuja, Nigeria, was observed with signs indicative of leptospirosis. Concurrent illness was observed in some kennel workers which had high titres of *leptospira* antibodies.

**Methods:**

An unmatched case-control study was conducted to identify associated factors. Fifteen cases and fifteen controls were recruited. A probable case was defined as any person working at the kennel with history of fever, chills, headache, myalgia with either conjunctivitis or sore throat between February and December 2009. Controls were healthy kennel workers within the same period. Inclusion criteria were any person meeting the definition (for both cases and controls). Kennel workers who were sick but did not fit into the case definition were excluded.

**Results:**

Bivariate analysis showed that wounds or cuts on either hands or legs of kennel workers during the period of the outbreak and contact with sewage at the kennel together (p = 0.001) was associated with leptospirosis among kennel workers.

**Conclusion:**

The findings revealed the importance of environmental hygiene in the prevention and control of leptospirosis. Sanitation and hygiene improvements were recommended.

## Introduction

Leptospirosis is a bacterial zoonosis that occurs worldwide. It is caused by spirochetes of the genus *Leptospira* that affects humans and some animals including rodents, ruminants, birds, dogs, reptiles, horses and amphibians [1]. Human infection results from contact with water or soil contaminated with urine of infected rats and other animals. Symptoms of acute leptospirosis in humans vary from fever, headache to myalgia, vomiting, conjunctivitis and diarrhoea. In complicated cases it may involve the liver, kidneys and vascular system [2]. Regarding dogs, symptoms include sudden onset of illness, fever, depression, anorexia, vomiting, conjunctivitis and muscular weakness. It may progress to hypothermia, profound depression, dyspnoea, muscular tremor, jaundice, heamatochezia, melaena, polyuria and to oliguria in advanced cases [1]. Greater risk of leptospirosis is reported in older dogs than less than one-year-old and in male dogs rather than females [3].

In Africa, approximately 154 human cases of leptospirosis occur annually. The disease is believed to be under-estimated in Nigeria with incidence of six human cases reported annually [4]. Canine leptospirosis is endemic in south-west Nigeria with *Grippotyphosa, Pomona* and *Bratislava* having higher prevalence than the old vaccinal serovars of *Canicola* and *Icterohaemorrhagiae* [5].

Between February and October 2009 an increase in the morbidity and mortality of dogs in a national kennel in Abuja, Nigeria, which had been operating for about three years, was observed with clinical signs indicative of leptospirosis. A total of 76 of the 117 dogs under observation were affected (attack rate (AR) = 65%), with case fatality rate (CFR) of 32%. The male to female ratio was 1.5:1. All ages, ranging from 5 months to 6 years (median age 2 years) were affected. All breeds were affected (Alsatian, Rottweiler, Boer boar, Labrador, Doberman, Pit bull, Terrier cross and Cocker spaniels), though Alsatians were the breed most affected with 45 animals (AR = 59%). Spectrum of clinical signs includes - anorexia, fever, bloody (frank/melena) diarrhoea, dyspnoea, vomition, oculonasal discharges and oedema of various body parts. All four of the canine blood specimens tested positive for *Leptospira interrogans* by enzyme-linked immunosorbent assay (ELISA). Illness was also observed in some kennel workers with symptoms associated with human leptospirosis within May and September 2009. Venous blood samples were collected from volunteer kennel workers to be confirmed by the laboratory in order to carry out an unmatched case-control study to identify associated factors for leptospirosis infection among kennel workers.

## Methods

**Study area and population:** The national kennel is situated in Nuwalege Village, Abuja. Abuja is located at 9°4'0'N latitude and 7°29'0"E longitude with a human population of 1 405 201 [6]. The kennel had undergone repair of the sewage system a month prior to the commencement of the investigation. It used to have some drain leakages and blocked sewage system which had both been repaired. An average of 2-4 rats (*Rattus rattus*) was sighted by the kennel workers at a single time within and outside the kennel. The kennel used to have problems with flooding during rainy season which had been corrected. The study populations comprised 63 workers at the national kennel in Abuja.

**Study design:** An unmatched case-control study involving 15 cases and 15 controls was conducted.

**Case definition:** A probable case was defined as any person working at the kennel with a history of symptoms fever, chills, headache and myalgia with either conjunctivitis or sore throat between February and December 2009. Controls were defined as healthy kennel workers with no obvious signs of disease. Inclusion criteria were any person meeting the definition (for both cases and controls), who were also seropositive to ELISA test kit (cases) and seronegative to ELISA (controls). Kennel workers who were sick but did not fit into the case definition were excluded from the study. The non-response rate was 35% and 44% among cases and controls respectively.

**Ethical considerations:** We obtained clearance from the Federal Ministry of Health and permission from the host organization as well as signed informed consent from all participants who were willing to participate in the study.

**Laboratory methods:** Venous blood sample was collected from ten volunteer case patients and ten volunteer controls. The blood samples were collected in vacutainer tubes containing anti-coagulants, preserved in ice-packs and sent to the laboratory section of the Veterinary Teaching Hospital at the University of Ibadan. The blood samples were tested for leptospirosis through the use of SmartVetComb™ Canine Leptospira antibody test kit which is a modified ELISA, which can be described as an enzyme labelled? dot assay’ that detects antibody levels in serum, plasma, or whole blood. The kit is not intended to distinguish the specific serotype but to determine antibody titres to different pathogenic serovars of *Leptospira interrogans (L. canicola, L. icterohaemorrhagiae, L. grippotyphosa, L. pomona)*. A positive result indicates current infection. The titre can be evaluated through the use of the CombScale™ and approximated to equivalent microscopic agglutination test (MAT) titre.

**Data collection:** A self administered structured questionnaire was used to obtain data on demographic factors and other potential risk factors based on reports and observations in the kennel, factors such as having wounds or cuts on either hands or legs within February and October 2009; contact with kennel sewage within the same period; contact with mud at work place within the same period; wading through flood water within the same period and engagement in extracurricular activities like swimming, farming, fishing, plumbing work and slaughtering of animals.

**Data analysis:** Data were entered and analyzed using Epi-Info version 3.3. Frequencies and odds ratios were determined and differences in frequencies of the considered variables between cases and controls were tested using Fisher’s exact test and exact confidence level. Level of significance was at 95% confidence interval. The association of risk factors with contracting leptospirosis was assessed by bivariate analyses.

## Results

**Laboratory result:** The blood samples of the ten volunteer case patients tested were positive at equivalent titre value of 1:200 each; while those of the ten volunteer controls were negative to *Leptospira interrogans*.

**Case-control:** The mean age of the cases and controls were 34 ± 5 years and 35.5 ± 6 years, respectively. About 94% and 87% of the cases and controls respectively were males. The mean year of service of both cases and controls were 1.7 ± 1.3 years and 1.4 ± 1.3 years respectively. A total of 87% and 67% of the cases and controls, respectively, were dog handlers. None of the cases had less than secondary education while 6.7% of controls had less than secondary education level. The month of onset for case workers was May and the last case was reported in September. Cases and controls were well balanced by age, gender, in job designation among dog handlers and veterinarians with the exception of nurses and administrative job as well as years of service (Table 1). Bivariate analyses revealed that having wounds or cuts on either hands or legs within February and October 2009 (OR = 12.0, 95% CI = 1.7 - 147.5) and contact with kennel sewage within the same period (OR = 15.0, 95% CI = 1.5 - 759) were significantly associated with acquiring leptospirosis among the kennel workers (Table 2). However, factors such as age, gender, level of education, years of service at the kennel, work designation, contact with flood and contact with mud during the period of February to October 2009, usage of at least hand gloves (among others - protective clothing, boots and face masks) among cases and controls, usage of at least protective clothing and at least gloves, protective clothing and protective boots were not significantly associated with leptospirosis among the kennel workers (Table 2). A total of 14 case workers (93.3%) and all the controls had their bath after finishing their work. All the cases and controls washed their hands before eating and after work schedule. Regarding the extracurricular activities for the cases, two (13.3%) were engaged in swimming and slaughtering of animals, one (6.7%) was engaged in fishing, whilst none was engaged in plumbing work as an extracurricular activity. None of the controls engaged in any of these activities. However, the differences in involvement in extracurricular activities had no significant association with acquiring leptospirosis. There was no significant association between leptospirosis and having exposure to either wounds or cuts on either hands or legs alone or having contact with sewage alone when compared with no exposure to both cuts and sewage. However, exposures to wounds or cuts on either hands or legs within February and October 2009 and contact with kennel sewage within the same period together was associated (p = 0.001) with leptospirosis among the kennel workers (Table 2).

**Table 1:** Demographic and occupational characteristics of kennel workers in Abuja, Nigeria 2009

Variables	Cases n = 15 (%)	Controls n= 15 (%)
**Age (years)**		
18-24	0 (0)	1 (6.7)
25-31	7 (47)	5 (33)
32-38	4 (27)	4 (27)
39-45	3 (20)	2 (13)
46-52	1 (6.7)	3 (20)
Mean age ± SD (years)	34 ± 5	36 ± 6
**Gender**		
Male	14 (93)	13 (87)
Female	1 (6.7)	2 (13)
**Number of years of service at the kennel**		
<1	4 (27)	8 (53)
1-2	4 (27)	1 (6.7)
> 2	7 (47)	6 (40.0)
**Mean service ± SD (years)**	**1.7 ± 1.3**	**1.4 ± 1.3**
Designation		
Dog handlers	13 (87)	10 (67)
Veterinarian	1 (6.7)	2 (13)
Nurses	1 (6.7)	0 (0)
Other Administrative staff	0 (0)	3 (20)
**Educational level**		
Primary	0 (0)	1 (6.7)
Secondary	8 (53)	6 (40)
Tertiary	7 (47)	8 (53)

**Table 2:** Association between leptospirosis and selected factors among kennel workers in Abuja, Nigeria 2009

Variable	Cases No. (%) (*n* = 15)	Controls No. (%) (*n* = 15)	OR (95% CI)	*p* value
Years of service at kennel				
< 1 years	4 (27)	8 (53)	0.3 (0.05-1.8)	0.26
1 + years	11	7		
**Work designation**				
Dog handlers	13 (87)	10 (67)	3 (0.4-39)	0.39
Non-Dog handlers	2	5		
**Level of education**				
Primary and secondary	8 (53)	7 (47)	1.3 (0.3-6.9)	1.00
Tertiary	7	8		
**Factors**				
Eating while working	1 (6.7)	3 (20)	0.3 (0.01-4.3)	0.60
Contact with sewage	14 (93)	7 (47)	15(1.5-759)	0.01**
Contact with flood	9 (60)	5 (33)	3 (0.5-17.3)	0.27
Contact with mud	11 (73)	5 (33)	5 (0.9-35.6)	0.07
Presence of wounds or cuts on either hands or legs	13 (87)	5 (33)	12.0 (1.7-147.5)	0.01**
No wounds or cuts on either hands or legs & No contact with sewage	0 (0)	4 (27)	Ref.	
Presence of wounds or cuts on either hands or legs only	1 (7)	4 (27)	∞‡ (0.021- 8)	NS*
Contact with sewage only	2 (13)	6 (40)	∞‡ (0.09- 8)	NS*
Presence of wounds or cuts on either hands or legs & Contact with sewage	12 (80)	1 (6)	∞‡ (2.7- 8)	0.001**
Use of at least hand gloves	10 (67)	11 (73)	0.7 (0.1-4.6)	1.00
Use of at least protective clothing	6 (40)	8 (53)	0.6 (0.1-3.1)	0.72
Use of at least gloves, boots and protective clothing	5 (33)	8 (53)	0.5 (0.08-2.4)	0.46
Engagement in extracurricular activities Farming	8 (53)	5 (33)	2 (0.42-13)	0.46

** Significant difference at p<0.05

‡ Division by 0.

* Not statistically significant. P>0.10

## Discussion

We reported associations between having wounds or cuts on either hands or legs during the period of the outbreak together with having contact with kennel sewage and contracting leptospirosis. This may be due to the fact that dogs which were the first to show signs of leptospirosis were shedding the leptospiral organism in their urine and subsequently to the kennel sewage [1, 2]. Thus, the kennel sewage could be the source of infection for the kennel workers while the wounds or cuts sustained on either hands or legs during this period could have served as the portal of entry of the leptospiral organism in the case workers, since almost all the case workers reported having their bath after close of work and washing of their hands before eating and after work schedule. However, our data had no conclusive evidence about association between presence of wounds or cuts on either hands or legs alone; or contact with sewage alone and contracting leptospirosis. It appears that either of these exposures is not sufficient enough alone as a factor to predispose to leptospirosis among kennel workers. This study, however, showed that both exposures together are sufficient factors to contracting leptospirosis among kennel workers. The month of onset of infection in dogs (February) preceded that of humans (May); the implication of this is that dogs could have acted as sentinels to warn of environmental contamination by leptospiral organisms. The dogs might have been exposed through activities of rats within and around the kennel. Rats are primary host of the Leptospiral organisms and they shed the bacteria in their urine [1, 7]. Sarkar et al. [8] and Sugnnan et al. [9] had also reported association between contact with sewage and leptospirosis in population-based studies. Sasaki et al. [10] and Nardone et al. [11] reported having wounds or cuts as a risk factor for leptospirosis in a population-based study.

The study revealed that the antibody level of volunteer cases tested were 1:200. The positive result in this particular test is an indication of current infection by *Leptospira*antigen as reported by the test kit manufacturer. The titre level of 1:200 indicates moderate infection to the *Leptospira* antigen. The test detects real time infection and not antibodies arising from vaccination. The test result established the presence of an on-going infection at the time of the investigation. *Leptospira interrogans* serovars such as *Canicola, Hardjo, Pomona, Icterohaemorrhagiae, Pyrogenes, Autumnalis and Grippotyphosa* have been reported in humans. However, serovars *Icterohaemorrhagiae, Canicola, Pomona* and *Grippotyphosa* serovars are commonly reported in dogs [1].

The age and gender composition of the cases had no significant association with leptospirosis. This may be as a result of the age similarity among the kennel workers. Similarly, higher percentages of the kennel workers are male. This finding differs from the results of Everald et al. [12] on investigation of some risk factors for severe leptospirosis in Barbados where the authors reported greater risk of leptospirosis in older aged men and in males. The similarity in age composition and greater male to female ratio of the workers in this kennel might have obliterated the possible effect of an association.

Factors such as level of education of respondents, years of services at the kennel, work designation, contact with flood and contact with mud had no significant association with contracting leptospirosis as a result of this study. This is similar to the findings of Sasaki et al. [10] who reported no association with leptospirosis and having contact with mud or freshwater. However, years of service, work designation, contact with flood and mud had also been shown in previous studies [8, 12, 13] to be significant factors for leptospiral infection. Association between leptospirosis and with contact with mud in this study might not be significant also due to the small cluster of cases and controls. In addition, flood water and mud that came into contact with the workers might not have been contaminated. The kennel had only been in operation for 3 years and may not be sufficiently long enough to observe the effects on variations in services.

Wearing of at least hand gloves, or at least protective clothing, or at least gloves, protective clothing and boots was not found to be protective in this study. This is similar to the findings of Everald et al. [12] who reported no significant association between leptospirosis and the use of protective clothing among agricultural workers. Regular and proper use of personal protective equipment might have been protective.

Leptospirosis is an occupational risk to veterinarians, miners, rice field workers, soldiers and other categories involved in outdoor activities. In addition, it is a recreational risk to swimmers, campers and rafters [1, 2]. However, our study revealed that engagement in extracurricular activities such as swimming, farming, fishing, plumbing work and slaughtering of animals had no significant association with leptospirosis. Studies involving large population have reported associations between these factors and leptospirosis [11, 12]. The reason for this observation may be caused by their professional delineation (low exposure to these factors).

### Study Limitations

The findings of this study cannot be extended to other kennel workers worldwide for dissimilarity in kennel services but may be extended to other kennel workers in Nigeria due to a similarity in operations. The study has limited sample size arising from constraints in obtaining suitable control groups. The disease could not be confirmed in the cases using the standard Microscopic agglutination Test (MAT), recruitment was based on exposure to infection. However, the identified associated factors could be useful in early diagnosis of infection in kennel workers and prevention of further human infection.

## Conclusion

This study underscores the importance of environmental hygiene in the prevention and control of leptospirosis. It also indicates the possibility of dogs acting as sentinels for leptospirosis. Wounds or a cut on either hands or legs together with contact with sewage was associated with leptospirosis among kennel workers in Abuja, Nigeria; but no conclusive evidence about either alone. It was recommended that sanitation and hygiene in kennel environment to be improved and immediate repair of any sewage problem. Regular and appropriate use of personal protective equipment (PPE) should be encouraged among kennel workers. If regular and appropriate use of PPE cannot be guaranteed, kennel workers with wounds or cuts on either arms or legs should not work in a risk situation. The findings of this study have implications for early diagnosis, treatment and prevention of leptospirosis among kennel workers. In addition, this study underscores the need for a multidisciplinary approach in the prevention and control of leptospirosis. Health education of kennel workers on identified activities related to the risk of infection of kennel workers is desirable.
